# Structured, proactive care coordination versus usual care for Improving Morbidity during Post-Acute Care Transitions for Sepsis (IMPACTS): a pragmatic, randomized controlled trial

**DOI:** 10.1186/s13063-019-3792-7

**Published:** 2019-11-29

**Authors:** Marc Kowalkowski, Shih-Hsiung Chou, Andrew McWilliams, Cathryn Lashley, Stephanie Murphy, Whitney Rossman, Alfred Papali, Alan Heffner, Mark Russo, Larry Burke, Michael Gibbs, Stephanie P. Taylor, On behalf of Atrium Health ACORN Investigators, Ryan Brown, Larry Burke, Shih-Hsiung Chou, Kyle Cunningham, Susan L. Evans, Scott Furney, Michael Gibbs, Alan Heffner, Timothy Hetherington, Daniel Howard, Marc Kowalkowski, Scott Lindblom, Andrea McCall, Lewis McCurdy, Andrew McWilliams, Stephanie Murphy, Alfred Papali, Christopher Polk, Whitney Rossman, Michael Runyon, Mark Russo, Melanie Spencer, Brice Taylor, Stephanie Taylor

**Affiliations:** 1 Center for Outcomes Research and Evaluation, Atrium Health, 1540 Garden Terrace, Suite 308, Charlotte, NC 28203 USA; 2 Department of Internal Medicine, Atrium Health, Charlotte, NC USA; 3 Ambulatory Care Management, Atrium Health, Charlotte, NC USA; 4 Transition Services, Department of Internal Medicine, Atrium Health, Charlotte, NC USA; 5 Division of Critical Care, Department of Internal Medicine, Atrium Health, Charlotte, NC USA; 6 Department of Emergency Medicine, Atrium Health, Charlotte, NC USA; 7 Division of Hepatology, Department of Internal Medicine, Atrium Health, Charlotte, NC USA; 8 Division of Palliative Care, Department of Internal Medicine, Atrium Health, Charlotte, NC USA

**Keywords:** Sepsis, Infection, Continuity of patient care, Patient navigator, Health services, Pragmatic clinical trial

## Abstract

**Background:**

Hospital mortality for patients with sepsis has recently declined, but sepsis survivors still suffer from significant long-term mortality and morbidity. There are limited data that support effective strategies to address post-discharge management of patients hospitalized with sepsis.

**Methods:**

The Improving Morbidity during Post-Acute Care Transitions for Sepsis (IMPACTS) study is a pragmatic, randomized controlled trial at three hospitals within a single healthcare delivery system comparing clinical outcomes between sepsis survivors who receive usual care versus care delivered through the Sepsis Transition and Recovery (STAR) program. The STAR program includes a centrally located nurse navigator using telephone counseling and electronic health record-based support to facilitate best-practice post-sepsis care strategies for patients during hospitalization and the 30 days after hospital discharge, including post-discharge review of medications, evaluation for new impairments or symptoms, monitoring existing comorbidities, and palliative care referral when appropriate. Adults admitted through the Emergency Department with suspected infection (i.e., antibiotics initiated, bacterial cultures drawn) and deemed, by previously developed risk-stratification models, high risk for readmission or death are included. Eligible patients are randomly allocated 1:1 to either Arm 1, usual care or Arm 2, STAR. Planned enrollment is 708 patients during a 6-month period. The primary outcome is the composite of all-cause hospital readmissions and mortality assessed 30 days post discharge. Secondary outcomes include 30- and 90-day hospital readmissions, mortality, emergency department visits, acute care-free days alive, and acute care and total costs.

**Discussion:**

This pragmatic evaluation provides the most comprehensive assessment to date of a strategy to improve delivery of recommended post-sepsis care.

**Trial registration:**

ClinicalTrials.gov, NCT03865602. Registered retrospectively on 6 March 2019.

## Background

Sepsis is a common, life-threatening condition defined by organ dysfunction due to a dysregulated response to infection [1]. Aggressive early sepsis identification and treatment initiatives have decreased hospital mortality for patients with sepsis [2–4]. As mortality rates have improved, there has been a growing recognition of the downstream effects of sepsis for the approximately 14 million annual sepsis survivors who encounter increased long-term mortality and morbidity across functional, cognitive, and psychological domains [4–10].

Currently, post-acute care resources are not adequate to address the needs of sepsis survivors [11–14]. Inadequate post-sepsis care strategies are reflected by the high rates of adverse outcomes after sepsis hospital discharge such as increased mortality risk and strikingly high rates of healthcare utilization, including a 90-day hospital readmission rate of 40% and over 3,000,000,000 USD in potentially preventable costs [15–19]. To address persistent morbidity and mortality for sepsis survivors, experts developed best-practice recommendations to guide delivery of post-sepsis care [20]. These recommendations are directed toward the specific challenges and sequelae following a sepsis hospitalization and include: identification and treatment of new physical, mental, and cognitive deficits; review and adjustment of medications; surveillance of treatable conditions that commonly lead to poor outcomes, including chronic conditions that may de-stabilize during sepsis and recovery; and focus on palliative care when appropriate. Implementation of these recommendations is hindered by a gap in understanding how to best integrate interventions into the complex and fragmented post-discharge setting (e.g., lack of provider time and patient engagement, limited access to care management, and insufficient institutional support) [21–26]. Furthermore, implementing these care recommendations requires health system investments, yet the effect of these investments on patient outcomes and costs has not been evaluated to date.

To evaluate the implementation and effectiveness of best-practice post-sepsis care recommendations, we developed a sepsis survivor transition program, in which a nurse facilitates implementation of recommended care practices and bridges care gaps through a program called Sepsis Transition and Recovery (STAR). The Improving Morbidity during Post-Acute Care Transitions for Sepsis (IMPACTS) trial is designed to test the hypothesis that implementation of the STAR program reduces 30-day readmission and mortality rates for high-risk patients with suspected sepsis compared to usual care alone.

## Methods

### Design

The IMPACTS study is a pragmatic, randomized controlled trial with two parallel groups being conducted at three tertiary care hospitals located within metro Charlotte, NC, USA to evaluate clinical outcomes for sepsis survivors receiving usual care versus care delivered through the STAR program following hospitalization. The STAR program is designed using the Chronic Care Model theoretical framework [27], which promotes care planning, active follow-up, and patient, provider, and community engagement, to increase adherence to best-practice recommendations and improve care coordination between hospital and post-acute care transitions during sepsis recovery. Consistent with a pragmatic study design, eligibility criteria are broad and study procedures are embedded into the context of routine care. This trial was approved by the Atrium Health (AH) Institutional Review Board (IRB) with a waiver of informed consent as this evaluation utilizes elements routinely collected in usual clinical practice and deemed to present minimal risk to study participants (IRB #01–19-24E; protocol version 1.0, date December 17, 2018). The trial is registered with ClinicalTrials.gov (NCT03865602), and the trial protocol adheres to the Standardized Protocol Items: Recommendations for Interventional Trials (SPIRIT) guidelines [28] (for SPIRIT checklist and figure, see Additional files 1 and 2) and the Pragmatic-Explanatory Continuum Indicator Summary 2 (PRECIS-2) domains for the design of pragmatic studies (for diagram of PRECIS-2 domains, see Additional file 3) [29].

### Study setting and population

**Fig. 1:**
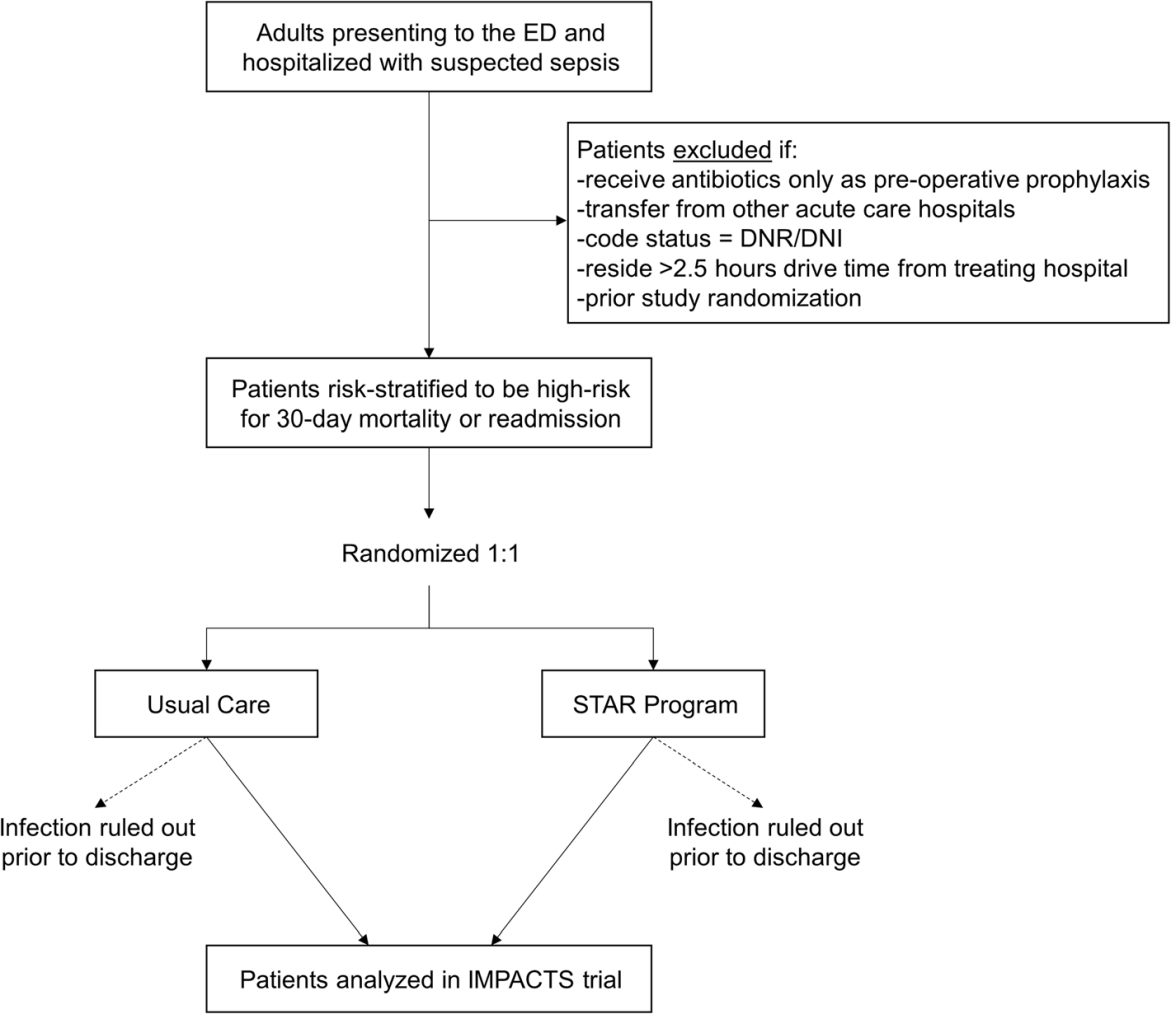
Patient flow diagram for participation in THE IMPACTS trial. The study population includes adults presenting to the Emergency Department (ED) who meet the following inclusion criteria: ≥ 18 years of age; oral or parenteral antibiotic or bacterial culture order within 24 h of ED presentation and either culture drawn first, antibiotics ordered within 48 h or antibiotics ordered first, culture ordered within 48 h; not discharged from the hospital at the time the daily list of eligible patients is generated each weekday morning; and deemed high risk for either 30-day readmission or mortality using risk-scoring models applied daily to real-time clinical data on acute and chronic factors. Patients are excluded based on receipt of prophylactic antibiotics only, hospital transfers, “do not resuscitate” or “do not intubate” (DNR/DNI) code status, distance of residence from treating hospital, and prior study randomization. Patients who have infection ruled out prior to hospital discharge are also excluded. *IMPACTS* Improving Morbidity during Post-Acute Care Transitions for Sepsis, *STAR* Sepsis Transition and Recovery

This trial will occur at three facilities within AH, one of the largest, integrated healthcare delivery systems in the United States. The study population is depicted in Fig. 1 and includes adults admitted to the hospital from the Emergency Department (ED) who meet the following inclusion criteria: ≥ 18 years of age; oral or parenteral antibiotic or bacterial culture order within 24 h of ED presentation and either culture drawn first, antibiotics ordered within 48 h or antibiotics ordered first, culture ordered within 48 h (adapted from Third International Consensus Definitions for Sepsis and Septic Shock criteria) [30]; not discharged from the hospital at the time the daily list of eligible patients is generated each weekday morning; and deemed high risk for either 30-day readmission or mortality, defined as a readmission risk probability ≥ 20% or mortality risk probability ≥ 10%.

Patients are excluded if they: receive antibiotics only as part of pre-operative prophylaxis; transfer from other acute care hospitals; have a code status documented as “do not resuscitate” or “do not intubate” within 24 h after admission (due to the general assumption of increased risk of exposure to less aggressive treatment); reside > 2.5 h drive time from the treating hospital (due to the maximum reach of the community paramedicine services leveraged within the STAR program and the general assumption that these patients may have less comprehensive utilization tracking within AH record systems for study outcomes); or have been previously randomized as part of this study. Because we initially identify patients with suspected infection in order to intervene with high-risk patients in near real time, we will re-evaluate eligibility based on infection status at the time of hospital discharge. Specifically, patients who have had an infection diagnosis ruled out during their hospitalization will be excluded for the purposes of analysis (i.e., rule-out documented in medical record, assessed by blinded, adjudicated chart review for both arms). All remaining eligible patients at the time of discharge will be included in analyses.

### Risk scoring and recruitment

Separate risk prediction scores are applied to assess 30-day hospital readmission and 30-day mortality using previously developed logistic regression models. Each model is derived from readily captured variables collected as part of routine clinical care during the first 24 h after ED presentation, such as physiologic measurements, laboratory values, basic sociodemographic characteristics, and personal medical history [31]. All model covariates are sourced from a patient’s clinical data during hospitalization and billing history at the time of hospital admission for near real-time application. Model performance was determined using 10-fold cross-validation (30-day mortality model discrimination: area under the receiver operator characteristic curve (AUC) = 0.85, negative predictive value (NPV) = 0.97; 30-day readmission model discrimination: AUC = 0.70, NPV = 0.89). High-risk features included older age, higher number of comorbidities (Charlson Comorbidity Index), hospital admission within the last 90 days, chemotherapy receipt within the last 90 days, respiratory agent receipt within the last 90 days, hyperlactatemia, hypotension, leukocytosis, and tachypnea. Risk probabilities between 0 and 1 are assigned to hospitalized patients for each outcome.

Each weekday (i.e., Monday–Friday), we generate an automated list of eligible patients at high risk for 30-day mortality or readmission, as identified by our previously developed risk models. Patients are randomly assigned to STAR or usual care arms. For those patients allocated to receive care via STAR, their information is sent by secure e-mail to the STAR navigator. For patients in both arms, data from the automated, daily patient list are sent to the study database for tracking via a computer-based process. At any point, patients may decline participation in STAR or any components of usual care.

### Trial interventions

Patients in the usual care group continue to receive usual care throughout their hospitalization and following discharge. Usual care elements are not prescribed but typically consist of: patient education and follow-up instructions at discharge; routine recommendations for follow-up visits with primary care providers; arrangements for home health services, transitional care, or care management follow-up based on each patient’s needs but not specifically tailored to the sepsis population; and, when necessary, discharge to post-acute skilled nursing facility (SNF) or acute rehabilitation settings but with no sepsis-specific follow-up. Consistent with the concept of a pragmatic trial, aspects of usual care are determined by treating clinicians independent of trial assignment.

Patients in the intervention arm receive care via the STAR program. The STAR program employs a centrally located nurse navigator who has clinical knowledge of sepsis and its cognitive and functional sequelae, core competencies in navigating transitions of care (e.g., facilitating communication, coordinating care, assessing/addressing barriers to care, providing patient education and practical resource information/referrals), and works as an extension of AH’s Transition Services within the Division of Hospital Medicine, which is a multidisciplinary team providing acute care support during the peri-discharge interval. We have previously published on the potential for Transition Services to improve 30-day readmission rates in patients with sepsis [32, 33]; this study seeks to extend the reach of those services through nurse navigation. All outreach from the STAR navigator occurs virtually (e.g., telephone, messaging, and electronic health record (EHR) systems) to provide proactive coordination and monitoring for patients. The targeted, evidence-based or best-practice care components include: identification of and referral for new physical, mental, and cognitive deficits; review and recommendation for adjustment of medications; surveillance of treatable conditions that commonly lead to poor outcomes; and referral to palliative care when appropriate (Table 1).

**Table 1:** Post-sepsis guidelines with the Sepsis Transition and Recovery (STAR) program task

Core component/evidence	Recommendation^a^	STAR task
Screen for new physical, mental, and cognitive deficits after sepsis		
Functional disability: patients aged ≥ 65 years develop one or two new functional limitations	– Prescribe structured exercise program – Referral to physical/cardiac/pulmonary rehabilitation as needed	Confirm functional assessment (physical therapy). Refer as needed
Swallowing impairment: of patients aged ≥ 65 years, 1.8% readmitted < 90 days for aspiration pneumonitis	– Screen for cough, dysphagia, weak voice – Referral to speech therapy as needed	Confirm screen and team aware. Refer as needed
Mental health impairment: prevalence for clinically significant anxiety 32%, depression 29%, and PTSD 44%	– Review details of hospital course (e.g., ICU diary) – Depression screen – Referral to peer support or behavioral health as needed	Mental health screen. Refer as needed
Review and adjust long-term medications		
Medication errors: errors of omission and commission occur in up to 25% of patients, depending on the medication	– Review antibiotic choice, dose, duration – Start/continue medications for comorbidities; adjust for BMI, etc. – Discontinue hospital medications without ongoing indication	Antibiotic stewardship Medication reconciliation Vitals/weight
Anticipate and mitigate risk for common and preventable causes of health deterioration		Routine virtual follow-up Schedule provider visits
Infection: of patients aged ≥ 65 years, 11.9% readmitted < 90 days for infection (6.4% for sepsis)	– Patient education about symptoms of sepsis, recurrence – Appropriate vaccination – Monitor for symptomatic improvement in index infection	Education Medication reconciliation Monitor symptoms
Heart failure exacerbation: of patients aged ≥ 65 years, 5.5% readmitted < 90 days for CHF	– Reassess beta blocker, diuretic, ACE-inhibitor dosing – Monitor volume status (fluid balance), recognizing dry weight may be decreased if muscle mass is lost	Medication reconciliation Vitals/weight Monitor symptoms
Acute renal failure: of patients aged ≥ 65 years, 3.3% readmitted < 90 days for acute renal failure	– Monitor renal function; laboratory testing as needed – Reassess need and dosages for renally cleared, nephrotoxic agents	Monitor symptoms Confirm CBC/BMP Medication reconciliation
COPD exacerbation: of patients aged ≥ 65 years, 1.9% readmitted < 90 days for COPD exacerbation	– Confirm/initiate appropriate controller inhalers – Appropriate vaccination – Review use of benzodiazepines/opioids	Monitor symptoms Medication reconciliation
Assess appropriateness for palliative care	– Palliative care screen/consult as indicated – Goals of care; educate on disease progression/ terminal	Discuss palliative care consult Goals of care

*ACE* angiotensin converting enzyme, *BMI* body mass index, *BMP* basic metabolic panel, *CBC* complete blood count, *CHF* chronic heart failure, *COPD* chronic obstructive pulmonary disease, *ICU* intensive care unit, *PTSD*, post-traumatic stress disorder

^a^Recommendations from Prescott and Angus [20]

At the initial telephone-based contact with the patient or caregiver during hospitalization, the STAR navigator introduces the STAR program and, in situations when the patient can participate, conducts a mental health screening using the Patient Health Questionnaire (PHQ)-2, with reflex administration of the PHQ-9 for positive PHQ-2 (i.e., ≥ 3 points on a scale of 0–6 points) [34]. The STAR navigator communicates the results to the patient’s attending physician. Additionally, the STAR navigator confirms consultations with physical therapy (with recommendation to consult delivered to the care team, if not in place), antibiotic stewardship (i.e., a coordinated program promoting appropriate antibiotic use with a pharmacist review of type and duration, with a review requested by the navigator if not completed), and an additional infectious disease specialist if ongoing systemic inflammatory response syndrome criteria are present more than 48 h after infection onset (i.e., at least two of the following clinical findings: body temperature < 36 °C or > 38 °C, heart rate > 90 beats/min, respiratory rate > 20 breaths/min, and white blood cell count < 4000/mm^3^ or > 12,000/mm^3^) [35, 36]. Finally, if the patient has a serious, chronic illness and either failure to improve after 5 days or a previous hospital admission in the last 60 days, the STAR navigator recommends a goals of care discussion led by the care team or a palliative care consultation [37]. Every 24–48 h during the remainder of the hospital stay, the STAR navigator reviews the patient’s electronic record, communicates with the patient or caregiver, and checks with the clinical case management team for updated discharge planning. Prior to discharge, the STAR navigator provides infection-specific education to the patient and caregiver, which also includes what to expect during transition from the hospital and written information on scheduled outpatient appointments and planned telephone touchpoints. The STAR navigator also reviews discharge orders and confirms inpatient pharmacist review of high-risk medications, including: appropriate indication if prescribed proton pump inhibitor, opioids, benzodiazapines, or antipsychotics; appropriate medications prescribed for chronic lung disease or chronic heart failure (e.g., inhaled corticosteroids for chronic lung disease; or diuretics, beta blockers, angiotensin-converting enzyme inhibitors, aspirin, or statins for chronic heart failure); and medication doses adjusted as needed if any new or worsening renal failure.

**Fig. 2:**
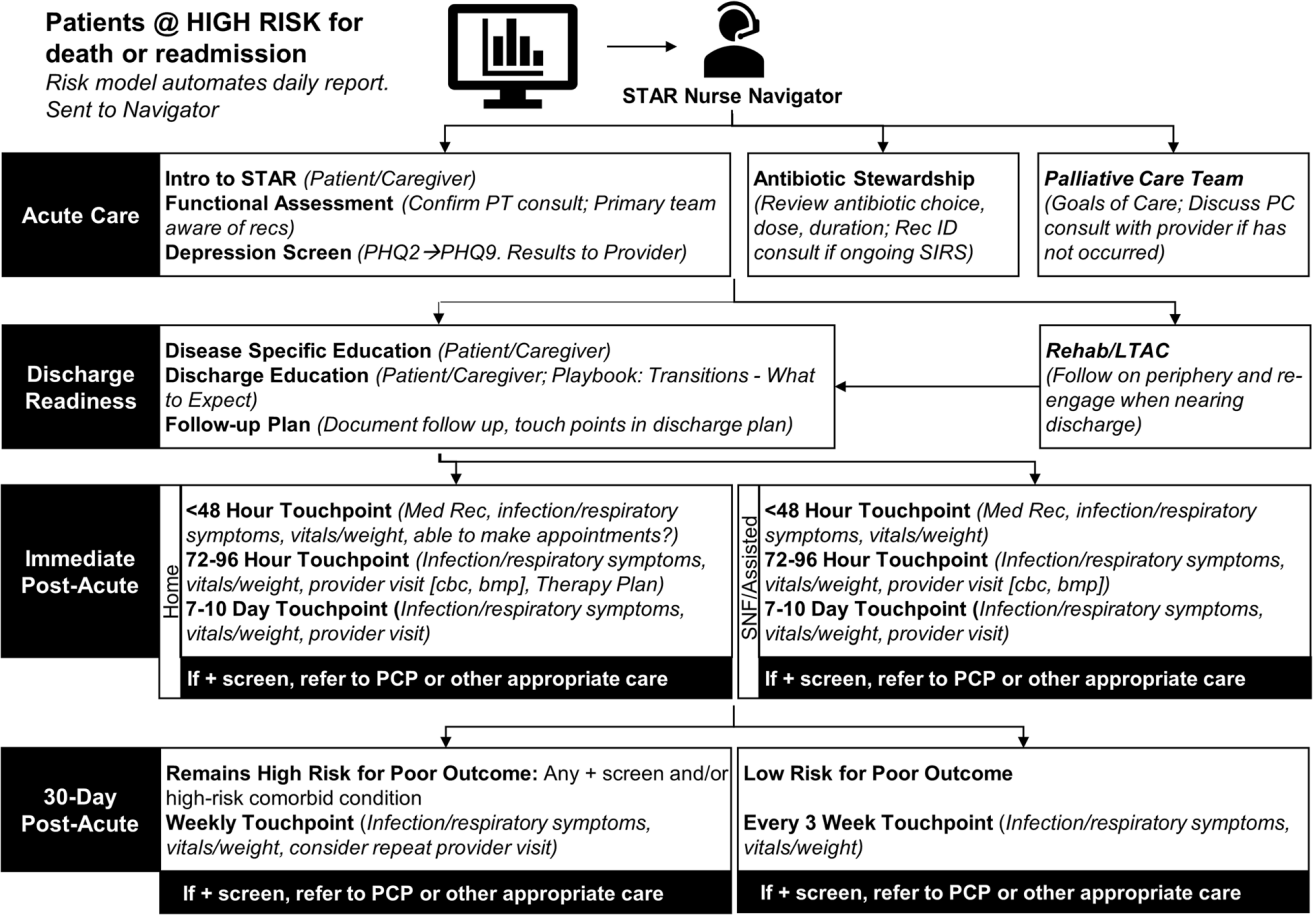
Sepsis Transition and Recovery (STAR) program description. The scheduled touchpoints for patients in the STAR program are depicted. Patients and caregivers are first introduced to the STAR program during hospitalization. Specific STAR program tasks to be performed during the acute care, discharge readiness, immediate post-acute, and 30-day post-acute intervals are summarized. bmp basic metabolic panel, cbc complete blood count, LTAC long-term acute care, Med Rec medical record, PC palliative care, PCP primary care provider, PHQ Patient Health Questionnaire, PT physical therapy, SIRS systemic inflammatory response syndrome, SNF skilled nursing facility

After discharge, the STAR navigator follows patients regardless of the discharge location (e.g., home, SNF) and remotely monitors via EHR-based and telephone-based review throughout the 30 days post hospital discharge (Fig. 2). Specifically, the STAR navigator provides telehealth monitoring at < 48 h, 72–96 h, and 7–10 days post discharge. Within 24–48 h of hospital discharge, the STAR navigator contacts the patient or caregiver for initial post-discharge medication reconciliation, with a review by the AH Transition Services pharmacist and confirmation that the patient has filled or received all medications needed. The STAR navigator coordinates with the patient’s primary care provider or AH Transition Services to address any outstanding medication needs. Also at this touchpoint, the STAR navigator monitors for fever (> 38 °C after recheck), new or worsening symptoms (e.g., dyspnea, diarrhea, or redness, swelling, or pain (for skin and soft tissue infection)), and new limitations in functional status (e.g., not out of bed, not eating). Concerns identified through proactive monitoring prompt a primary care provider contact for follow-up within 24 h. If the primary care provider cannot be reached after one attempt, the navigator contacts AH Transition Services to coordinate either a virtual visit with a physician facilitated by community paramedicine or an in-person physician visit [32]. The STAR navigator also meets at least weekly with the AH Transition Services Medical Director to review ongoing cases and coordinate additional support as needed. Additional touchpoints at 72–96 h and 7–10 days post discharge include similar elements (i.e., medication reconciliation, targeted symptom monitoring, vitals and weight checks, confirmation that the patient can make scheduled outpatient appointments) and response (i.e., coordinating follow-up with primary care or AH Transition Services within 24 h for concerning issues). If a provider visit has been completed, the STAR navigator reviews any visit notes, available laboratory values (e.g., complete blood count, basic metabolic panel), and documented therapy plan. After the first 10 days following hospital discharge, the STAR navigator maintains weekly telehealth touchpoints with patients who remain at high risk for poor outcome, defined as any previous positive screen or high-risk comorbid condition (e.g., chronic lung disease, heart failure) [10, 38–40]; and one additional third-week touchpoint with patients considered low risk after the first 10 days post discharge. Each of these post-acute touchpoints include targeted symptom monitoring, vitals and weight check, and escalation to an additional outpatient provider visit within 24 h if there are concerns. Any identified concerns again prompt an attempt to contact the primary care provider followed by AH Transition Services. The STAR program intervention completes 30 days post hospital discharge.

### Treatment allocation

Eligible patients are randomly allocated 1:1 to receive usual care or the STAR program using a computer-based randomization tool and maximally tolerated imbalance procedure with up to 10% allowable deviation to maintain similar comparison group sizes while limiting predictability of future treatment assignments [41]. Allocation concealment is achieved as the randomization is a fully automated process. However, it is not feasible to blind clinicians or patients to treatment. Due to resource limitations that allowed for only one full-time STAR navigator, the total daily number of patients randomized is constrained to include up to six patients each weekday (from the daily list of eligible patients, sorted by time of presentation). The randomization constraint is reevaluated on a biweekly basis and adjusted as needed to match the STAR navigator’s capacity.

### Data collection

Navigators document completion of STAR workflow processes in the patient’s EHR through the care management electronic documentation form, and then data are exported into a secure, research database (REDCap) [42]. All clinical and outcomes data are collected directly from the AH EHR system and Enterprise Data Warehouse (EDW). Data for study population description are presented in Table 2. Data are collected on a near real-time basis and include physiologic measurements (e.g., mean arterial pressure), laboratory values (complete blood count, basic/comprehensive metabolic panel, lactate), basic sociodemographic characteristics (e.g., age, gender, race, marital status, insurance status), past medical history (e.g., comorbidities, prior healthcare use, medication history), hospital procedures (e.g., mechanical ventilation), and organizational variables (e.g., hospital).

**Table 2:** IMPACTS study patient characteristics

Characteristic	Usual care (*n* =)	STAR program (*n* =)
Age at admission (years)		
Median (IQR)	–	–
> 65 years	–	–
Gender		
Male	–	–
Female	–	–
Race		
White	–	–
Black	–	–
Other	–	–
Marital status		
Married	–	–
Separated or divorced	–	–
Single	–	–
Widowed	–	–
Insurance		
Medicare	–	–
Medicaid	–	–
Private	–	–
Self pay/other	–	–
Comorbid conditions		
Chronic lung disease	–	–
Chronic renal disease	–	–
Diabetes	–	–
Heart failure	–	–
Malignancy	–	–
Myocardial infarction	–	–
Peripheral vascular disease	–	–
Charlson Comorbidity Index, median (IQR)	–	–
Number of hospital admissions < 6 months, median (IQR)	–	–
Index hospitalization organ dysfunction measures		
Mean arterial pressure (mmHg), median (IQR)	–	–
Mean arterial pressure < 70 mmHg, *n* (%)	–	–
Creatinine (mg/dl), median (IQR)	–	–
Creatinine > 2.0 mg/dl, *n* (%)	–	–
Bilirubin (mg/dl), median (IQR)	–	–
Bilirubin > 2.0 mg/dl, *n* (%)	–	–
Platelets (cells/μl), median (IQR)	–	–
Platelets < 100 cells/μl, *n* (%)	–	–
Lactate (mmol/L), median (IQR)	–	–
Lactate ≥ 2.0 mmol/L, *n* (%)	–	–
Mechanical ventilation during index hospitalization	–	–
Vasopressor receipt during index hospitalization	–	–

*IMPACTS* Improving Morbidity during Post-Acute Care Transitions for Sepsis, *IQR* interquartile range, *STAR* Sepsis Transition and Recovery

### Study outcomes

The primary outcome is a composite, dichotomous endpoint of all-cause mortality or unplanned hospital readmission assessed 30 days post index hospital discharge (Table 3). This combined outcome is ideally suited to our pragmatic study design because the elements are uniformly captured from data contained in the AH EDW, minimizing non-differential assessment, outcome misclassification, and missing data. Additionally, mortality and hospital readmission are widely regarded as patient-centered outcomes, and rates for both adverse outcomes remain high after sepsis hospitalization [43–45]. Finally, readmission rates have recently declined secondary to focused initiatives [46]. However, some data suggest increased mortality during the same interval [47], indicating the importance of measuring mortality and readmission rates in combination. Mortality is defined as any date of death documented in the AH EDW from index hospital presentation to within 30 days of index hospital discharge, including events from national death record data fed monthly into the EDW [48]. Readmission is defined as any unplanned inpatient or observation encounter to any of 47 AH hospitals within the 30 days following index hospital discharge. Both inpatient and observation status hospitalizations count toward the readmission outcome, because either status represents an adverse event important to patients and healthcare systems. This information is captured from encounter data in the AH EDW and has been previously extracted by the study team.

**Table 3:** IMPACTS primary and secondary outcomes

Clinical and cost outcomes	Usual care (*n* =)	STAR program (*n* =)
Primary outcome		
30-day all-cause mortality or hospital readmission	–	–
Secondary outcomes		
30-day all-cause mortality	–	–
30-day hospital readmission	–	–
30-day sepsis/infection-related hospital readmission	–	–
30-day chronic lung disease-related hospital readmission	–	–
30-day heart failure-related hospital readmission	–	–
30-day acute renal failure-related hospital readmission	–	–
30-day emergency department visits	–	–
30-day acute care-free days alive	–	–
30-day acute care costs	–	–
30-day total healthcare costs^a^	–	–
90-day all-cause mortality	–	–
90-day hospital readmission	–	–
90-day sepsis/infection-related hospital readmission	–	–
90-day chronic lung disease-related hospital readmission	–	–
90-day heart failure-related hospital readmission	–	–
90-day acute renal failure-related hospital readmission	–	–
90-day emergency department visits	–	–
90-day acute care-free days alive	–	–
90-day acute care costs	–	–
90-day total healthcare costs^a^	–	–

*IMPACTS* Improving Morbidity during Post-Acute Care Transitions for Sepsis, *STAR* Sepsis Transition and Recovery

^a^Only analyzed among Medicare-insured subgroup

The following secondary outcomes are assessed at 30 and 90 days after hospital discharge: all-cause mortality; all-cause, unplanned hospital readmission; cause-specific hospital readmissions with primary diagnoses (based on *International Classification of Diseases, 10th revision* diagnosis codes) related to sepsis or common infection (i.e., sepsis (A40–41, R65.20–21), pneumonia (J13–18), urinary tract infection (N30, N34, N39.0), skin and soft tissue infection (L00–08)), chronic lung disease (J40–47), heart failure (I50), and acute renal failure (N17); ED visits; acute care costs for services received at ED, inpatient, or observation encounters to any AH facility; total healthcare costs for inpatient and outpatient services received at any AH or outside system hospital or provider clinic (only in the subgroup of patients enrolled in a Medicare Shared Savings Plan with complete claims data tracked in AH EDW); and acute care-free days alive, defined as the sum of days alive without inpatient, observation, or ED encounters (rounded to a full day for any day with acute care utilization) during the interval after discharge. First, the total potential follow-up time is calculated as the number of days from index discharge to the earliest date of death or 30 (or 90) days post discharge (patients who die during their index hospitalization have 0 days alive). Each potential follow-up day is categorized as either an acute care day or an acute care-free day, based on any inpatient, observation, or ED encounter on that day. Total acute care-free days alive are calculated as the total potential follow-up time minus the number of acute care days during the 30 (or 90) days after index hospital discharge.

To provide additional context to understanding STAR implementation, important process measures are tracked in both groups, including: functional assessment or physical therapy consult [49, 50]; mental health assessment by PHQ-2 or PHQ-9 [51]; referrals to physical therapy or outpatient rehabilitation, speech therapy, and behavioral health [52–55]; early outpatient follow-up (i.e., completed outpatient primary care follow-up (or attendance at an AH Transition Services clinic) within 7 days of discharge) [56–60]; and documented medication reconciliation in the EHR during the 30 days post discharge [61, 62]. Because sepsis may occur in the setting of long-standing illness and declining health [63–65], we also measure the quality of end-of-life care, including place of death (i.e., hospital or other location) and the proportion of patients who received a palliative care consult, completed care preferences documentation, and were discharged to a hospice.

### Statistical analysis

Primary analyses will follow an intent-to-treat approach such that patients will be analyzed based on the group to which they were initially randomized after exclusion of patients with infection ruled out during their hospitalization. We will assess the balance in the distributions of baseline covariates for patient factors (e.g., age, comorbidities), acuity (e.g., organ dysfunction, mechanical ventilation), length of stay, and discharge disposition (e.g., SNF, rehabilitation, home) between study groups. Comparisons of the two groups will be made using univariate analyses such as the *t* test and chi-square test.

The primary composite outcome, 30-day readmission and mortality, will be compared between the two arms using logistic regression. We will present the effects of the STAR program on the incidence of the composite readmission and mortality outcome as odds ratios and 95% confidence intervals. In addition to the primary intent-to-treat analysis, and since there is significant overlap between general AH Transition Services and the STAR program services integrated within AH Transition Services, we will conduct a modified intent-to-treat analysis excluding usual care patients who attended the AH Transition Services clinic during the 30 days after hospital discharge. Based on historical data, we anticipate an approximately 10% referral rate to AH Transition Services in the usual care group. Finally, we will complete a per-protocol analysis to compare patients in the STAR intervention arm who are eligible at hospital discharge and complete the 30-day STAR program to patients who are eligible at hospital discharge and receive usual care.

Secondary acute care and cost outcomes and process measures will be evaluated using the same approach. We will test different distribution parameters to determine the optimal distribution family for each model and outcome variable (e.g., gamma distribution for costs, Poisson distribution for acute care-free days). Any substantial changes to the study processes will be documented, discussed with investigators at monthly meetings, and incorporated into analyses of study outcomes. All hypothesis tests will be two sided and data will be analyzed using SAS Enterprise Guide v7.1 (Cary, NC, USA).

### Subgroups

In addition to the overall study population, primary and secondary outcomes will be compared between the usual care and STAR intervention arms within several clinically relevant subgroups, including: chronic comorbidity burden (i.e., Charlson Comorbidity Index ≥ 5 or < 5); acute severity of index hospitalization (i.e., septic shock or no shock, defined by fluid-resistant hypotension requiring vasopressors and hyperlactatemia (> 2 mmol/L)) [66]; patients who survive the index hospitalization; and Medicare patients over age 65 years (due to the presence of complete claims data on these patients and the additional financial incentives and programs aimed at reducing total and post-acute care spend in this population).

### Data Safety and Monitoring Board

Due to the low risk associated with study participation, no interim analyses are planned to evaluate for potential harm related to the intervention and a Data Safety and Monitoring Board is not convened.

### Sample size calculation

This study is designed to detect a 25% relative reduction in the primary outcome, the composite rate of 30-day readmission and mortality, which is reasonable given prior literature suggesting that between 22% and 42% of hospital readmissions after sepsis are preventable, including data from a secondary analysis of the subgroup of sepsis patients in our previous transitions of care trial (relative risk = 0.49, 0.24–0.97) [33]. Based on the historical data, the control group is estimated to have a 40% combined readmission and mortality rate. Group sample sizes of 354 in the STAR group and 354 in the usual care group achieve 80% power (α = 0.05) to detect a 10% absolute reduction between the group proportions (i.e., 25% relative reduction). Under the alternative hypothesis, the proportion in the STAR group is assumed to be 30%.

### Missing data

We do not anticipate substantial missing data because all outcomes are routinely collected variables and utilization is broadly captured within our large integrated system. Values for patients who do not have healthcare utilization or mortality records during the study follow-up interval are assumed to be null. While utilization may occur outside AH, this is not expected to be a major limitation because of the AH market share and accessibility. Specifically, AH operates three large hospitals in Cabarrus and Mecklenburg Counties and more than 40 hospitals in the region overall. Additionally, any utilization occurring outside the system is anticipated to be non-differentially distributed between groups and thus to impact treatment groups equally. Further, internal historical data indicate that nearly 75% of high-risk patients are Medicare-insured (i.e., Medicare Shared Savings Plan beneficiaries). For these patients, we will have complete healthcare claims within and outside AH facilities during the study interval, as captured through participation in the local AH-managed Accountable Care Organization. We will conduct subgroup analyses within this Medicare-insured population and will use these data to explore missing data patterns that can be adjusted using pattern-mixture methods in sensitivity analyses.

## Discussion

The IMPACTS trial evaluates a multifaceted, patient-centered transitional care program for sepsis survivors that focuses on addressing both their immediate post-discharge needs as well as the long-term challenges in managing the downstream effects of sepsis. The intervention centers on a sepsis nurse navigator who aims to empower the patient or caregiver to manage the patient’s health condition beyond the intervention period. Evidence of an effective intervention and real-world implementation does not currently exist to guide health system decision-making and investments aimed at improving morbidity and mortality in sepsis survivors. Upon completion, this trial will provide comprehensive data on the effectiveness of delivering best-practice post-sepsis care through proactive care coordination to improve outcomes for high-risk patients. Results demonstrating better clinical outcomes for patients in the STAR program would provide evidence that structured care coordination integrated into robust transitions programs can be a useful strategy to improve the immense long-term healthcare burden of sepsis, while null findings would suggest that hospital resources may be better directed toward evaluating other post-sepsis care strategies to improve patient outcomes.

We considered several potential study design approaches while planning the IMPACTS trial. Ultimately, we concluded that a pragmatic, patient-level, randomized clinical trial was the most feasible and appropriate evaluation strategy. One central element in our decision-making process was our determination that a patient-randomized approach would not be subject to substantial unplanned cross-over that could bias outcome results. Specifically, physicians will not be able to direct usual care patients to the STAR program because this process will be determined through our automated list generation and randomized allocation. The STAR program will not accept physician referrals during the study interval. Additionally, we determined that the pragmatic design would effectively leverage our robust data systems and objective collection of clinical and outcomes data to facilitate a rigorous, real-world evaluation of post-sepsis care.

We intentionally developed broad infection criteria to initially define adults with suspected serious infection or sepsis, with the additional application of our high-performing risk stratification models to objectively narrow the population to those at the highest risk of poor outcomes and with the potential to benefit most from added support. We anticipate that this approach will include a representative sample of patients with different chronic and acute problems. Because of the expected heterogeneity, we have defined a priori several subgroups of interest to further evaluate possible differential effects of the STAR program (e.g., patients with high comorbidity burden).

There are some limitations to this study. The results may not be generalizable to all sepsis survivors because study participants are enrolled from a single healthcare system. However, the inclusion of three heterogeneous hospitals within the healthcare system increases the applicability of our findings. Second, we did not protocolize the usual care arm, thus care provided to patients in this group may be variable or change over time. While this may increase noise in determining the true treatment effect, it is consistent with the concept of a pragmatic study design. The STAR program was developed in partnership with a multidisciplinary team of sepsis stakeholders and designed to be implemented and evaluated in a real-world setting. If effective, this program would offer a timely solution for hospitals facing potential financial penalties for higher-than-expected readmission rates and looming alternate payment models such as bundled payments and shared savings programs for Accountable Care Organizations, which creates incentives to deliver care in less intensive settings during the 90 days after hospitalization.

### Trial status

The IMPACTS trial is an ongoing pragmatic randomized controlled trial evaluating clinical outcomes data for adult patients who receive care through the STAR program versus usual care during and after hospitalization for suspected sepsis. The first patient was enrolled on January 28, 2019. Protocol version 1.0 was applied (date December 17, 2018). Recruitment is anticipated to be completed by the end of 2019.

## Supplementary information

**Additional file 1.** SPIRIT 2013 Checklist: recommended items to address in a clinical trial.

**Additional file 2.** SPIRIT Schedule of enrollment, interventions, and assessments.

**Additional file 3.** Pragmatic-Explanatory Continuum Indicator Summary 2 (PRECIS-2) tool describing the assessment of nine domains used to inform pragmatic trial development.

## Data Availability

Datasets generated during the current study will not be shared.
