# Supplementary material for: Structured, proactive care coordination versus usual care for Improving Morbidity during Post-Acute Care Transitions for Sepsis (IMPACTS): a pragmatic, randomized controlled trial
Source: Trials. 2019 Nov 29;20:660. doi: 10.1186/s13063-019-3792-7 (PMC6884908; doi:10.1186/s13063-019-3792-7)
Supplement: Supplementary file 2 — Additional file 2. SPIRIT Schedule of enrollment, interventions, and assessments. [file 13063_2019_3792_MOESM2_ESM.docx]

Additional file 2. Schedule of enrollment, interventions, and assessments

|  | **STUDY PERIOD** | | | | |
| --- | --- | --- | --- | --- | --- |
|  | **Enrolment** | **Allocation** | **Post-allocation** | | |
| **TIMEPOINT**** | ***-t_1_*** | **0** | ***t_1_*** | ***t_2_*** | ***t_3_*** |
| **ENROLLMENT** |  |  |  |  |  |
| **Initial Eligibility** | X |  |  |  |  |
| **Allocation** |  | X |  |  |  |
| **Eligibility assessment at time of discharge (infection diagnosis ruled out yes/no)** |  |  | X |  |  |
| **INTERVENTIONS** |  |  |  |  |  |
| ***STAR Program*** |  |  | X | X |  |
| ***Usual Care*** |  |  | X | X |  |
| **ASSESSMENTS** |  |  |  |  |  |
| ***Demographics and Clinical Variables*** | X | X |  |  |  |
| ***Primary and Secondary Outcomes (Mortality, Readmission, ED visits, acute care days, and cost)*** |  |  |  | X | X |
| ***Process measures (functional and mental health assessments, early outpatient followup, medication reconcilliation)*** |  |  |  | X |  |

***-t_1_ and t_0_:** occurs within 72 hours of a patient’s presention to the Emergency Department (i.e., maximum time allowed for satisfying criteria for infection on admission). Eligibility assessment is automated using data elements available in the Electronic Health Record (EHR). Treatment allocation to Sepsis Transition and Recovery (STAR) program intervention or usual care is automated and occurs following eligibility evaluation. Baseline demographic and clinical data are also collected at this time point.

** **t_1_:** patient elibility is reassessed at the time of hospital discharge and individuals with infection ruled out are excluded.

***** t_2_:** 30 day: mortality, readmissions, Emergency Department (ED) visits, acute care days, and acute care and total healthcare costs are collected at this time point. Process measures are also collected.

****** t_3_:** 90 day: mortality, readmissions, Emergency Department (ED) visits, acute care days, and acute care and total healthcare costs are collected at this time point.
