# Supplementary figures and images for: Structured, proactive care coordination versus usual care for Improving Morbidity during Post-Acute Care Transitions for Sepsis (IMPACTS): a pragmatic, randomized controlled trial
Source: Trials. 2019 Nov 29;20:660. doi: 10.1186/s13063-019-3792-7 (PMC6884908; doi:10.1186/s13063-019-3792-7)

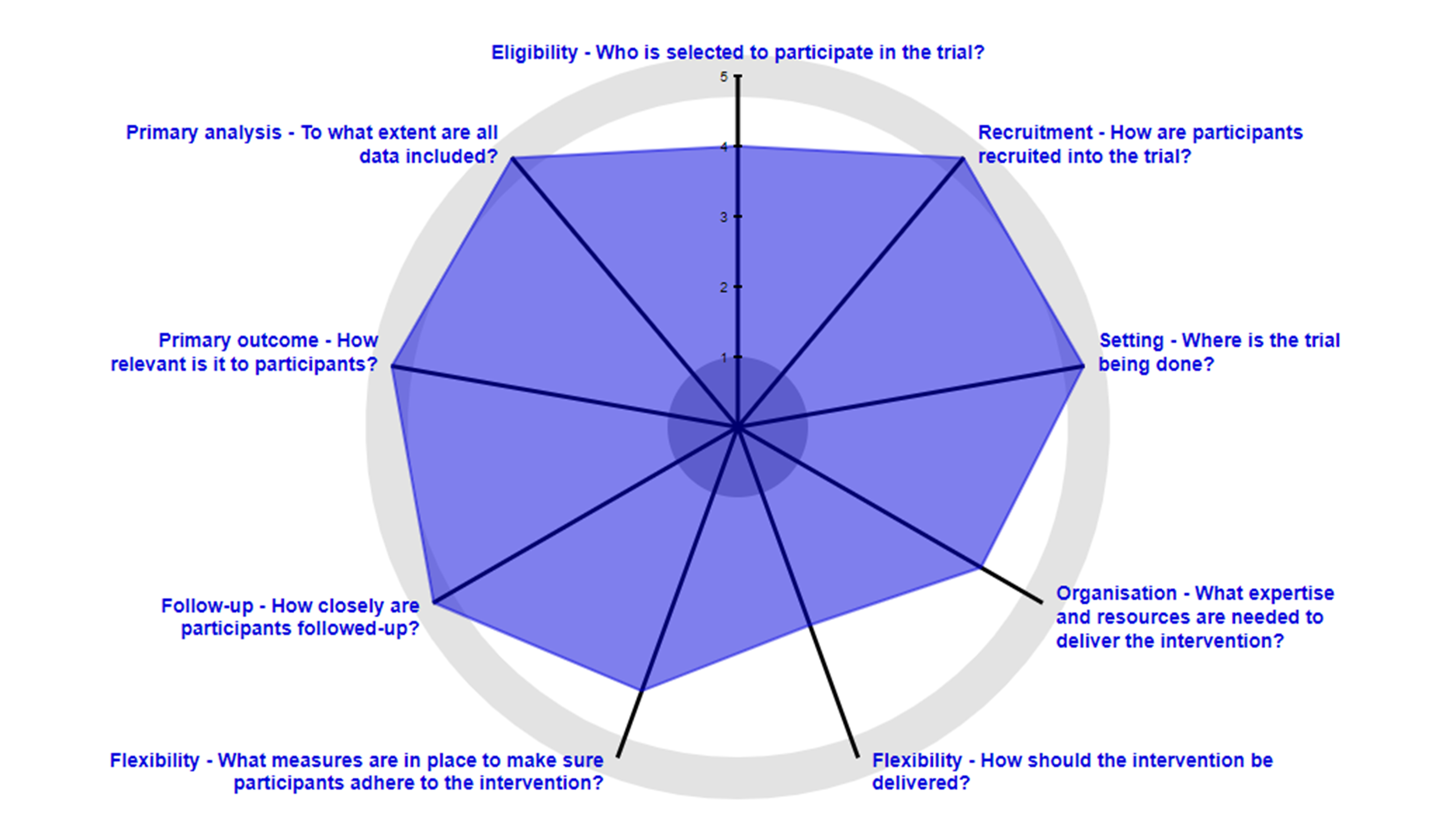

Supplement: Supplementary file 3 — Additional file 3. Pragmatic-Explanatory Continuum Indicator Summary 2 (PRECIS-2) tool describing the assessment of nine domains used to inform pragmatic trial development. [file 13063_2019_3792_MOESM3_ESM.bmp]
